# Transcriptomic signatures of NK cells suggest impaired responsiveness in HIV-1 infection and increased activity post-vaccination

**DOI:** 10.1038/s41467-018-03618-w

**Published:** 2018-03-23

**Authors:** Margaret C. Costanzo, Dohoon Kim, Matthew Creegan, Kerri G. Lal, Julie A. Ake, Jeffrey R. Currier, Hendrik Streeck, Merlin L. Robb, Nelson L. Michael, Diane L. Bolton, Nicholas J. Steers, Michael A. Eller

**Affiliations:** 1 U.S. Military HIV Research Program, Walter Reed Army Institute of Research, 503 Robert Grant Ave., Silver Spring, MD 20901 USA; 2 Henry M. Jackson Foundation for the Advancement of Military Medicine, 6720A Rockledge Dr., Bethesda, MD 20817 USA; 3 Virus Diseases Branch, Walter Reed Army Institute of Research, 503 Robert Grant Ave., Silver Spring, MD 20901 USA; 4 Institute for HIV Research, University Hospital Essen, University Duisburg-Essen, 45147 Essen, Germany; 5 Division of Nephrology, Columbia University Medical Center, 1150 St. Nicholas Ave., New York, NY 10032 USA

## Abstract

Natural killer (NK) cells limit viral replication by direct recognition of infected cells, antibody-dependent cellular cytotoxicity (ADCC), and releasing cytokines. Although growing evidence supports NK cell antiviral immunity in HIV-1 infection, further knowledge of their response is necessary. Here we show that NK cells responding to models of direct cell recognition, ADCC, and cytokine activation have unique transcriptional fingerprints. Compared with healthy volunteers, individuals with chronic HIV-1 infection have higher expression of genes commonly associated with activation, and lower expression of genes associated with direct cell recognition and cytokine stimulation in their NK cells. By contrast, NK cell transcriptional profiles of individuals receiving a modified vaccinia Ankara (MVA) vectored HIV-1 vaccine show upregulation of genes associated with direct cell recognition. These findings demonstrate that targeted transcriptional profiling provides a sensitive assessment of NK cell activity, which helps understand how NK cells respond to viral infections and vaccination.

## Abstract (web summary)

Natural killer (NK) cells are important for eliminating cells under stress or infected by virus, and may have a function in anti-HIV immunity. Here the authors show that different NK-activating stimuli induce distinct transcriptional fingerprints in human NK cells that are analogous to changes caused by HIV vaccination or chronic infection.

## Introduction

Natural killer (NK) cells are innate effector lymphocytes that kill virus-infected or -transformed cells and represent an important component of the human immune system^1^. Poised to respond rapidly to infection, NK cells possess an array of stimulatory and inhibitory receptors such as the following: killer cell immunoglobulin-like receptors (KIRs); C-type lectin receptors, natural cytotoxicity receptors (NCR), and Toll-like receptors (TLR)^2,3^. Given evidence that NK cells regulate adaptive immunity and develop memory like features to specifically recognize microbial antigens, NK cells possess both innate and adaptive qualities^4^. The diversity of the NK lineage suggests an evolutionary mechanism in protection from viral infection and highlights a gap in our understanding of the function of these cells in the human immune response.

As a matter of convention, the neural cell adhesion molecule (CD56) and the low-affinity antibody-binding receptor Fcγ-receptor IIIa (CD16A) are used to classify immunomodulatory and cytotoxic NK cells^5^. Cytotoxic NK cells are able to sense altered expression of major histocompatibility complex (MHC) and kill those cells through receptors designed to monitor normal human leukocyte antigen (HLA) expression, a process known as the altered or “missing self” response^6^. NKG2D is an activating C-type lectin able to recognize cellular stress ligands on the cell surface, such as MICA, MICB, and ULBP1-4 that accumulate in response to infection or transformation^7^. Concomitant missing class I MHC antigens and expression of surface stress ligands results in a robust NK cell killing response that is replicated in in vitro models through stimulation with K562 cells and other activating cell lines^8,9^. Strong NK effector activity is also observed in response to signaling through CD16 mediated antibody-dependent cellular cytotoxicity (ADCC)^10–12^ allowing for a hybrid adaptive feature through antigen-specific recognition. One in vitro model of ADCC is performed by coating CEM.NKR.CCR5 cells, a cell line resistant to NK cell lysis, with antigens of interest and in the presence of antibodies to those antigens and NK cell function or target lysis is quantified. Additionally, viral infections induce a milieu of inflammatory and immunomodulatory cytokines, such as interleukin (IL)-2, IL-12-p70, IL-15, and IL-18 that are also able to activate and promote NK cell functional activity^13–15^.

The contribution of NK cells in combating human immunodeficiency virus (HIV) infection has been appreciated more since genetic association studies revealed KIR genes (*KIR3DL1* and *KIR3DS1*), in combination with certain HLA genotypes, are linked to protection from HIV infection and disease progression^16,17^. Further evidence of the impact of NK cell-mediated immune pressure is found in the HIV-1 viral genome, where sequence polymorphisms are found in association with certain KIR alleles^18^. NK cell response to HIV-1 in vivo remains unknown although in vitro data show the ability to respond to autologous infected CD4 T cells directly^19–21^ or the activation of NK cells through ADCC^22–24^. In fact, results from the RV144 HIV vaccine trial suggest that NK-mediated ADCC is a correlate of protection^25^. Indeed, NK cells possess diverse capacity to respond to a variety of pathologic and inflammatory conditions, including HIV-1, yet more information is needed to better understand the underlying framework for their effector function and how these cells may impact outcome. With this combined information, opportunities to direct and exploit NK cell antiviral immunity to target HIV have exponentially grown^17^.

Here we explore NK cell-mediated immune regulation at the protein and transcript level in healthy donors, HIV-1 chronically infected individuals, and healthy HIV-1 vaccine recipients. We find modest differences in cell surface marker expression by flow cytometric analysis within NK cells performing three distinct processes: a direct cell cytolysis model; an ADCC model; and response to inflammatory cytokine stimulation in vitro. However, gene expression analysis reveals exclusive differences in NK cells executing HIV-specific ADCC versus cytokine stimulation or direct cell cytolysis of target cells. Further evidence of NK cell activity is observed directly ex vivo in chronically HIV-1-infected individuals as well as in response to modified vaccinia Ankara-Chiang Mai Double Recombinant (MVA-CMDR) vaccination. Utilizing cell sorting and transcriptional profiling to identify unique transcripts and distinct biological pathways can unveil potential mechanisms by which NK cells control HIV-1 infection, which may inform how to better engage these cells through vaccination.

## Results

### Characterization of functional NK cells by flow cytometry

**Fig. 1:**
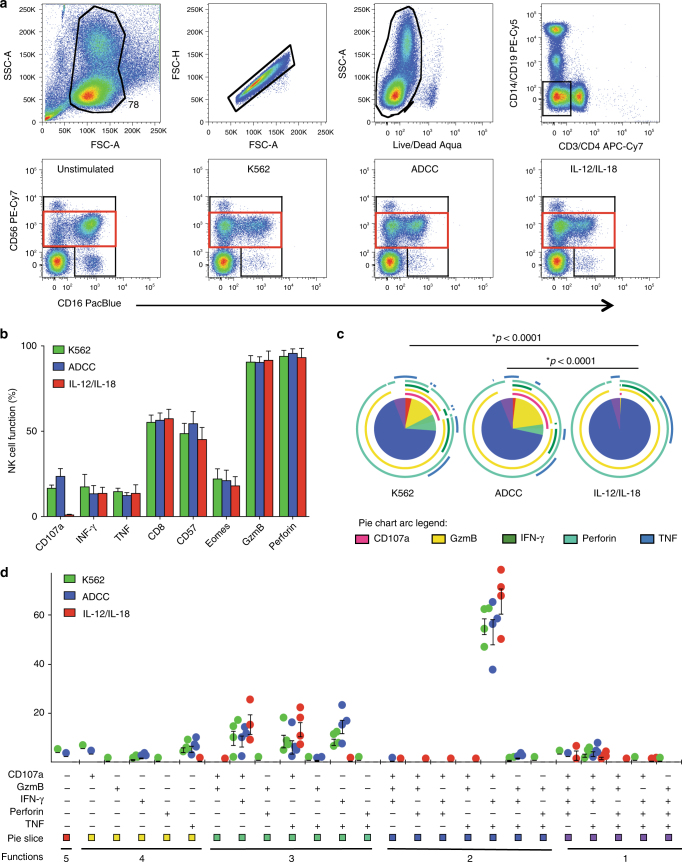
Frequency of responding NK cells across multiple stimulation conditions. **a** Top row: overall successive gating strategy demonstrates initial broad gating on forward and side scatter to include large lymphocytes. Forward area and height are used to discriminate single cells followed by identification of viable cells. Monocytes and B cells are excluded using CD14 and CD19, and T cells are excluded using CD3 and CD4. Bottom row: CD56 and CD16 are used to identify NK cells and changes in CD16 expression across stimulation conditions. Black gate is the total NK population and red gate is CD56 dim population. **b** A univariate analysis of the frequency of CD107a, INF-γ, TNF, granzyme B, perforin, and eomesodermin expression from the total NK cell population as well as CD57 and CD8 expression are shown. **c** Pie charts of classic Boolean analysis of five functional markers measured (CD107a, IFN-γ, TNF, granzyme B, and perforin). Comparison of pies and nonparametric partial permutation tests (Monte Carlo simulation) are performed to determine statistical significance between pies (*p*-value < 0.0001)^69^. **d** Graph of classic Boolean analysis of five functional markers measured. Four healthy subjects from four independent experiments in each stimulation condition are included. CD107a, IFN-γ, and TNF are mock subtracted (i.e., stimulated condition − unstimulated result). Black bars represent the standard error mean (SEM) for each stimulation condition calculated as the variance of the sampling distribution obtained, equal to the variance of the population divided by the sample size

NK cell function was assessed using a 14-color flow cytometry panel, as previously described^23^, to identify changes in bulk NK cell receptor expression and function of NK cells responding to various in vitro stimulation conditions. Immunofluorescently stained cells were studied to determine functional commonalities and differences between in vitro stimulation with the MHC-devoid K562 cell line to simulate direct cell recognition, an ADCC mimic (gp120-coated CEM.NKR.CCR5 cells, in the presence of HIV-IG) and inflammatory cytokine stimulation with IL-12 and IL-18 (Fig. 1). This study focused on the more abundant, mature, and functionally responsive CD56^dim^ NK cell population for functional interrogation^5,26^ (Fig. 1a, red box). Expression of CD16, quantified by the median fluorescent intensity (MFI), was lower across all stimulation conditions compared to the unstimulated control, consistent with literature suggesting downregulation of CD16 in response to stimuli^23,27–29^. Robust activation of NK cells, calculated as total expression of CD107a, interferon (IFN)-γ, and tumor necrosis factor (TNF), was observed with an average 30%, 30%, and 8% of NK cells responding to K562, an ADCC mimic, and cytokine stimulation, respectively. Univariate analysis showed no significant variation in IFN-γ, TNF CD8, CD57, eomesodermin, granzyme B, and perforin protein expression across the three stimulation conditions (Fig. 1b). However, upregulation of CD107a was different between the cell-based stimulation (both K562 and ADCC) and cytokine stimulation (Fig. 1b). Of note, CD8 and CD57 had higher surface expression on CD107a^+^ cells while eomesodermin, granzyme B, and perforin were expressed at lower levels on these cells, across all three stimulation conditions, compared to unstimulated cells. Combinatorial expression was evaluated in the CD56^dim^ compartment for markers CD107a, INF-y, TNF, granzyme B, and perforin, to determine functional differences, across stimulation conditions (Fig. 1c, d). Statistically significant differences were observed between K562 and cytokine stimulation as well as ADCC and cytokine stimulation (both *p* < 0.001, comparison of pies, nonparametric partial permutation tests (Monte Carlo simulation)). This was largely driven by the expression of CD107a, as cytokine stimulation does not induce degranulation of NK cells.

Mature and differentiated CD56^dim^CD57^+^ NK cells are reported to be more functional, post stimulation, compared to CD56^dim^CD57^−^ NK cells^30–32^. In order to assess this phenomenon within our cohort we compared functional responses of CD56^dim^CD57^−^ and CD56^dim^CD57^+^ NK cells, post stimulation, via intracellular cytokine staining (supplementary Fig. 1). When comparing CD107a and INF-γ expression, CD56^dim^CD57^−^ NK cells had slightly lower expression levels of these markers compared to CD56^dim^CD57^+^ NK cells in K562 and ADCC stimulation, although it did not reach statistical significance. Approximately twofold less CD56^dim^CD57^+^ NK cells produced INF-γ in response to cytokine stimulation, which is consistent with previous reports (supplementary Fig. 1a)^31^. Furthermore, a Boolean analysis of the six functional markers (CD107a, IFN-γ, TNF, granzyme B, perforin, and eomesodermin) showed no differences in total function between CD56^dim^CD57^−^ and CD56^dim^CD57^+^ NK cells (supplementary Fig. 1b). Collectively, this analysis demonstrates the difficulty in discriminating NK cell function across different response mechanisms by measuring intracellular and cell surface markers.

### Transcriptional signatures in functional NK cells

**Fig. 2:**
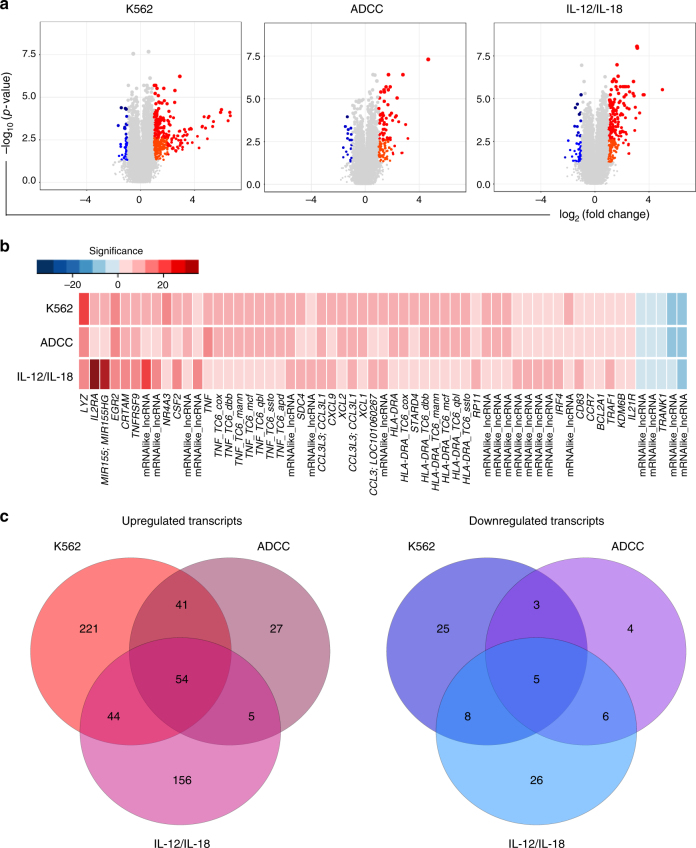
Whole-transcriptome analysis of functional NK cells. **a** Volcano plots representing upregulated and downregulated transcripts of functional NK cells in response to different stimulation conditions. Size of each data point is calculated as −log_10_(*p*-value) × log_2_(FC), *p*-value cutoff *p* < 0.05, fold change cutoff > 2 or <1/2. Red dots represent upregulated transcripts, blue dots represent downregulated transcripts. **b** Heat map comparison of common gene expression significance values (−log_10_(*p*-value)) across three stimulation conditions. **c** Venn diagrams displaying upregulated (red) and downregulated (blue) transcripts of functional NK cells across three stimulation conditions

In an effort to define unique and common signatures of NK cell activity that was not detected at the protein level, due the constraints of flow cytometry, we studied the entire transcriptome of NK cells. Due to downregulation of CD16 within the CD56^dim^CD16^bright^ compartment^23^, healthy donor CD56^dim^CD16^dim/neg^ cells, post stimulation were stained and sorted for gene expression analysis (supplementary Fig. 2). Human transcriptome array (HTA) technology was used to investigate expression profiles of responding NK cells across the three stimulation conditions in six donors (Fig. 2). Approximately 912 transcripts were differentially expressed in responding NK cells compared to unstimulated cells (Fig. 2a). K562 stimulation resulted in the most dramatic changes, where 221 transcripts were uniquely upregulated in NK cells involved in direct cell cytolysis. Twenty-seven transcripts were uniquely upregulated in NK cells performing ADCC and 156 transcripts were uniquely upregulated in response to cytokine stimulation (>2-fold change, and *p* < 0.001, Wilcoxon rank-sum test, supplementary data 1).

The transcriptional fingerprint (uniquely upregulated transcripts) of NK cells responding to K562 stimulation include the following: *BCAT1*, *CCNA2*, *CDC6*, *CENPN*, *CLIC2*, *DLGAP5*, *INSIG1*, *KIAA0101*, *NR4A2*, *NUF2*, *NUSAP1*, *PRC1*, *TPX2*, and *TXN*, with fold change increases in the range of 2.12–7.73 and *p*-value < 0.02 (Wilcoxon rank-sum test). The transcriptional fingerprint of NK cells responding to cytokine stimulation include the following: *AGK*, basic leucine zipper ATF-like transcription factor (*BATF*), *CCND2*, *CCR1*, *CD274*, *CD44*, *CDK6*, *DDX21*, *GZMB*, *HK2*, *HUWE1*, *IFNG*, *IL12RB2*, *IL18R1*, *MAP3K8*, *NDFIP2*, *NFKB1*, *PIM1*, *PIM2*, and *SLAMF7* with fold change increases in the range of 2.84–8.68 and *p*-value < 0.04 (Wilcoxon rank-sum test). The transcriptional fingerprint of NK cells responding to ADCC include *BCL2*, *GBE1*, *GPI*, *IGF2R*, and *OSBPL3* with fold change increases in the range of 2.09–2.67 and *p*-value < 0.01 (Wilcoxon rank-sum test). Recognizing the fact that some models of ADCC may not reflect triggering exclusively through CD16 and because of the requirement of NKG2D to achieve optimal levels of antibody-dependent cytolysis in our model^33^, we also tested an additional model of ADCC using the murine mastocytoma cell line P815, coated with monoclonal anti-human FCγRIIIa to discriminate signaling through CD16 (supplementary data 3). There were 17 common upregulated transcripts between the two models of ADCC and 80 unique transcripts specifically upregulated by GP120-coated CEM.NKR.CCR5 cell stimulation.

Upregulation of transcripts within all three stimulation conditions (common transcripts) reveal a signature of overall NK cell activation and inhibition encompassing multiple mechanisms (Fig. 2b). These common transcripts include *BCL2A1*, *CCL3*, *CCR7*, *CRTAM*, *CSF2*, *EGR2*, *HLADR*, *IL21R*, *IL2RA*, interferon regulatory factor 4 (*IRF4*), *KDM6B*, *LYZ*, *MIR155*, *NR4A3*, *PKM*, *PRDX1*, *SERPINB9*, *TNF*, *TNFRSF9*, *TNFAIP*, and *TRAF1* with fold change increases in the range of 2.07–7.52 and *p*-value < 0.01 (Wilcoxon rank-sum test, supplementary data 1). Overlap and uniqueness in gene expression was recognized between the three conditions, as displayed in the Venn diagrams (Fig. 2c). All upregulated and downregulated transcripts are listed in supplementary data 1 and 2.

We next performed a principal component analysis (PCA) of the gene expression data from NK cells across stimulation conditions. Distinct patterns were observed within upregulated transcripts for all conditions also found within downregulated transcripts with the exception of K562 and ADCC, which has some pattern overlap. For upregulated transcripts, principal component 1 (87.12%), principal component 2 (5.98%), and principal component 3 (1.63%) sufficiently explain variance of the overall data. Interestingly, transcripts *TFPI*, *BCAT1*, *HMGCS1*, *PTTG1*, *PRC1*, *CENPN*, *PPA1*, *CDC45*, *TPX2*, and *CLIC2* are most influential in PC1 and are involved in cell cycle, homeostasis pathways, and direct cell recognition. For downregulated transcripts, principal component 1 (91.7%), principal component 2 (2.59%), and principal component 3 (1.16%) explain the overall data variability. Interestingly, transcripts *C3AR1*, *ENPP5*, *SLAMF6*, *TRANK1*, *SMIM14*, *USP28*, *ATP841*, *PTPLAD2*, *DDX60*, and *SORL1* are most influential in PC1 and are involved in immune activation, cytoplasmic vesicle transport, and cell division.

### Gene Set Enrichment Analysis

**Fig. 3:**
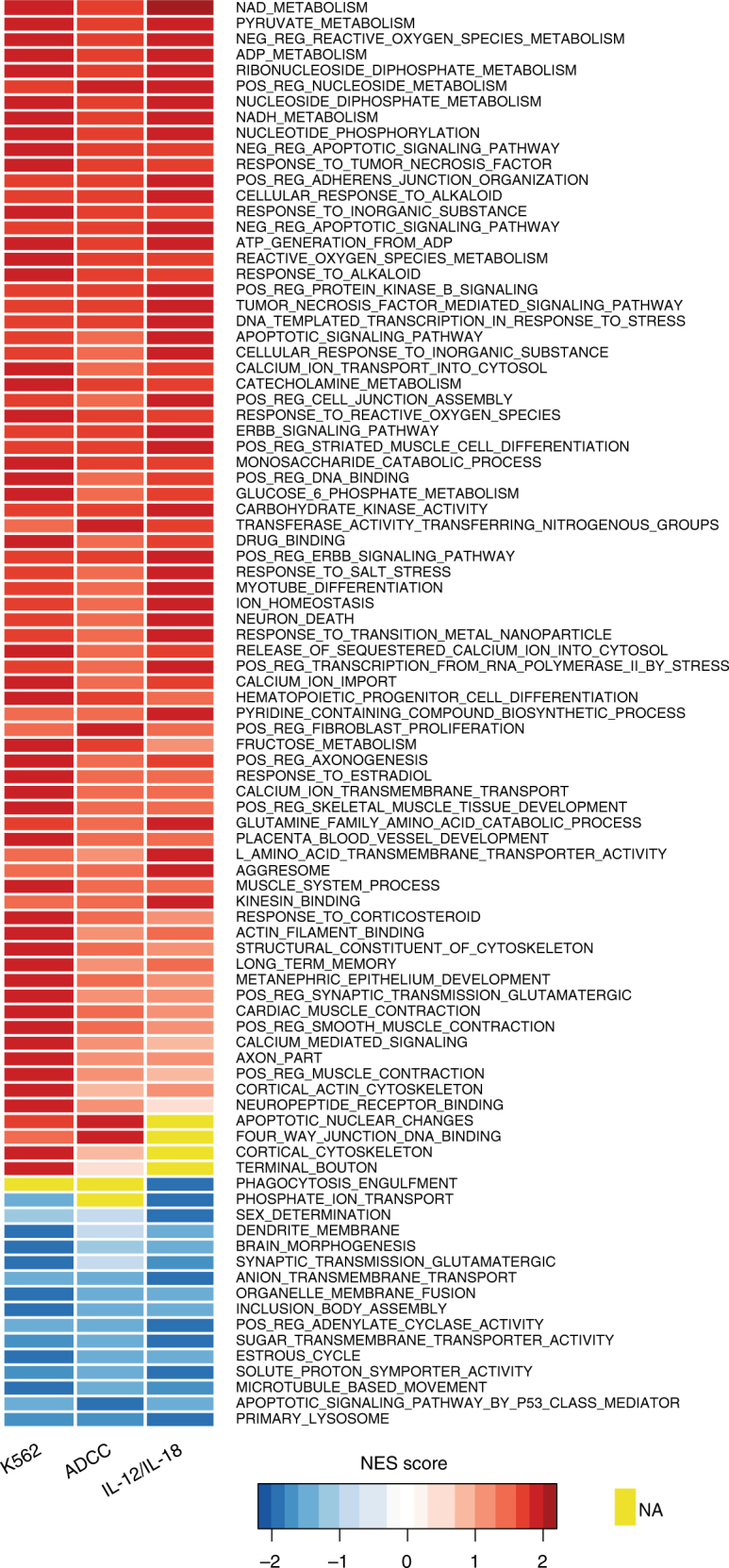
Identification of Gene Ontology (GO) pathways in responding NK cells. Heat map of GO pathways involved in NK responses across stimulation conditions, ranked by sum of normalized enrichment scores (NES) in the range of 1.8> or <−1.8. Transcriptional analysis from six healthy subjects from two independent experiments is represented. Yellow filled boxes indicate that the pathway was not identified

To examine biological pathways these differentially expressed transcripts are involved in, we next performed a Gene Set Enrichment Analysis (GSEA) using the Gene Ontology (GO) gene set to identify associations within each stimulation condition. The cutoff value for each pathway was a normalized enrichment score (NES) 1.8> or <−1.8. GSEA revealed top-ranked common pathways, which include chemokine receptor binding, chemokine activity, and G-coupled protein receptor binding (Fig. 3). Top-ranked pathways within K562 stimulation include ADP, glucose, pyruvate and NADH metabolic process, cortical cytoskeleton, and negative regulation of apoptotic signaling with NES values ranging from 1.8 to 1.96 and false discovery rate (FDR) *q*-value < 0.05. Top-ranked pathways within ADCC include apoptotic nuclear changes, fructose and nicotinamide adenine dinucleotide (NAD) metabolic process, and negative regulation of intrinsic apoptotic signaling with NES values ranging from 1.80 to 1.85 and FDR *q*-value < 0.1. Top-ranked pathways within cytokine stimulation include NAD and pyruvate metabolic process, positive regulation of protein kinase b signaling, and amino-acid transmembrane transporter activity with NES values ranging from 1.8 to 2.02 and FDR *q*-value < 0.01. All pathways, NES values, and FDR *q*-values are listed in supplementary data 4.

### Gene expression at single-cell resolution

**Fig. 4:**
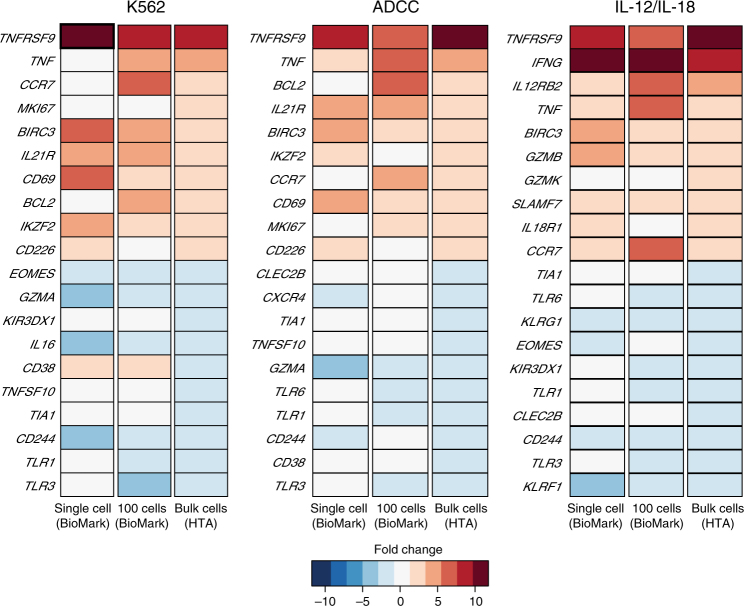
Gene expression of responding NK cells at single-, 100-cell, and whole-transcriptome levels. Gene expression heat map displaying top 10 upregulated and top 10 downregulated of 96 pre-selected genes involved in NK responses across stimulation conditions, ranked by fold change. Each column represents expression at the single-, 100-, and bulk-cell levels. Data represented are from three donors and three stimulation conditions, K562, ADCC, and IL-12/IL-18. Expression threshold (ET) values were calculated based on CT values (40-CT) obtained from the qPCR data and fold change was determined by comparison of ET values

Due to the heterogeneous nature of NK cells, we further interrogated whether the gene expression patterns of NK cells are representative of the entire CD56^dim^CD16^dim/neg^ population or reflective of a smaller subset. We employed single-cell index sorting and targeted transcriptional analysis of 96 genes at the single-cell and 100-cell levels (supplementary data 5) (derived from previous studies) to answer this question. Marked differences were observed when comparing relative gene expression of all 96 genes at the single-cell, 100-cell, and bulk-cell levels (supplementary Fig. 3). Interestingly, *CCL4*, which encodes beta chemokine MIP-1B, and *IL2A*, the gene encoding the alpha chain of the IL-2 receptor, gene expression was upregulated and detected only at the single- and 100-cell levels (supplementary Fig. 3). Therefore, exploring genes at the single- and 100-cell resolution reveals biologically relevant gene expression patterns. The top 10 upregulated and top 10 downregulated genes were determined across the three cell levels (Fig. 4), and noticeably, TNF receptor super family 9 (*TNFRSF9*) and Baculoviral IAP repeat containing 3 (*BIRC3*) were upregulated at all three cell levels and across all three stimulation conditions (Fig. 4). These genes are classified as commonly expressed genes in activated NK cells. Remarkably, Toll-like receptor 1 (*TLR1*), *CD244*, and *TIA1* are downregulated across all stimulation conditions. Albeit, *TIA1* is only downregulated at the bulk level, while *TLR1* and *CD244* are also downregulated at the 100-cell level, and none are downregulated at the single-cell level. Genes for NK cell receptors KLRF1 (NKp80) and CD244 (2B4) are downregulated in cytokine stimulation, in bulk cells, 100 cells, and at the single-cell level, which is consistent with previously published reports^34^.

### Gene expression analysis of NK cells directly ex vivo

**Fig. 5:**
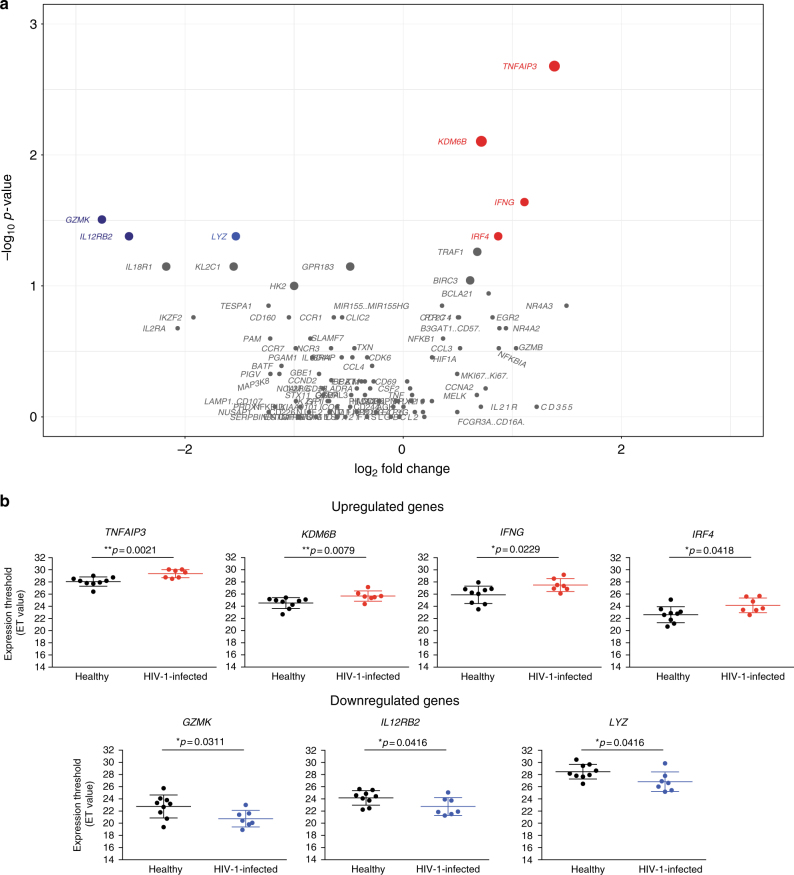
Gene expression of activated NK cells in chronically infected HIV individuals. **a** Volcano plot displaying upregulated (red), downregulated (blue), and stably expressed (gray) pre-selected genes, involved in NK cell-mediated responses. Size of each data point is calculated as −log_10_(*p*-value) × log_2_(FC), *p*-value cutoff *p* < 0.05, fold change cutoff > 2 or <1/2. **b** Expression threshold (ET) values plotted for top up (red)- and downregulated (blue) genes between healthy volunteers (black) and HIV-1 chronically infected individuals (red or blue). ET values were calculated based on CT values (40-CT) obtained from the qPCR data. Transcriptional analysis from nine healthy subjects and seven HIV-1 chronically infected individuals from two independent experiments is represented

Utilizing data from the whole-transcriptome analysis of CD56^dim^CD16^dim/neg^ NK cells in healthy donors we developed a panel of 96 genes within the transcriptional fingerprints related to common NK cell activation, direct cell cytolysis, ADCC, and cytokine stimulation (supplementary data 6). This targeted panel was used to identify transcriptional fingerprints related to CD56^dim^CD16^dim/neg^ NK cell function in HIV-1 chronic infection (supplementary data 7). NK cell transcriptional activity in HIV-1 chronically infected individuals (*n* = 7) compared to healthy individuals (*n* = 9) reveal that four genes are significantly upregulated and three genes significantly downregulated (Wilcoxon rank-sum test, Fig. 5a, b). Upregulated genes include *TNFAIP3*, *KDM6B*, *IFNG*, and *IRF4*, while downregulated genes include *GZMK*, *IL12RB2*, and *LYZ* with 1.64- to 2.82-fold change decreases and *p*-values ranging from 0.002 to 0.04 (Wilcoxon rank-sum test, Fig. 5a, b). These data suggest that NK cells in chronic HIV-1 infection demonstrate a transcriptional profile linked with overall NK cell activation. Interestingly and not so surprisingly, we observe a significant decrease in gene transcript-associated cytolytic content and cytokine receptor expression.

**Fig. 6:**
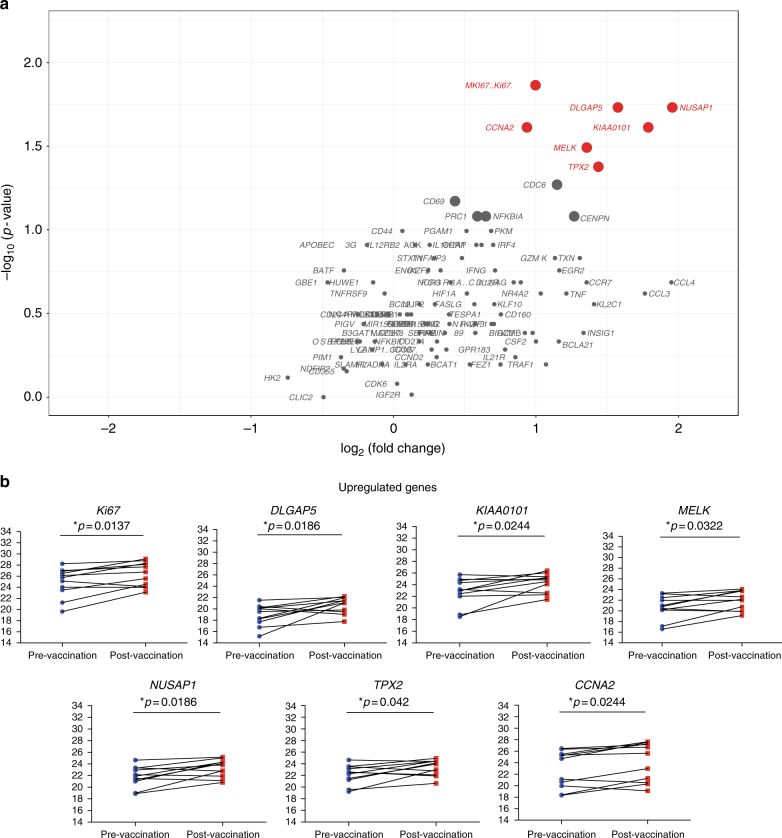
Gene expression of activated NK cells in vaccinated individuals. **a** Volcano plots displaying upregulated (red) and stably expressed (gray) pre-selected genes, involved in NK cell-mediated responses to MVA vaccination. Size of each data point is calculated as −log_10_(*p*-value) × log_2_(FC), *p*-value cutoff *p* < 0.05, fold change cutoff > 2 or <1/2. **b** Expression threshold (ET) values plotted for top upregulated genes between pre-vaccination group (blue) and post-vaccination group (red). ET values were calculated based on CT values (40-CT) obtained from the qPCR data. Transcriptional analysis from 11 vaccinees from two independent experiments is represented

Thereafter, we examined targeted gene expression of CD56^dim^CD16^dim/neg^ NK cells in a cohort of vaccinated individuals (*n* = 11) participating in a Phase I Study DNA primed (env and gag) and boosted by MVA-CMDR (HIV-1env/gag/pol) (supplementary data 8). We compared the differential gene expression profiles in 11 healthy individuals primed with PennVaxG DNA (env and gag) and boosted with MVA-CMDR containing HIV-1 CM235 env and CM240 gag/pol. Pre-vaccination and 7 days post MVA boost time points were chosen to determine gene expression in responding CD56^dim^CD16^dim/neg^ NK cells (Fig. 6a, b). Transcripts most significantly upregulated are *MKI67*, *NUSAP1*, *TPX2*, *KIAA0101*, *CCNA2*, *MELK*, and *DLGAP5* with 1.91- to 3.88-fold change increases and *p*-values ranging from 0.01 to 0.04 (Wilcoxon signed-rank test, Fig. 6a, b). All of these genes are associated with cell cycle and replication pathways. Most importantly, the majority of genes (6/7) are associated with the transcriptional fingerprint of direct cell cytolysis. These data demonstrate that NK cell activity is modulated after vaccination with the live viral vector MVA and this modulation is associated with the direct cell recognition and cytolysis.

### Gene expression patterns in CD57^+^NKG2C^+^ NK cells

**Fig. 7:**
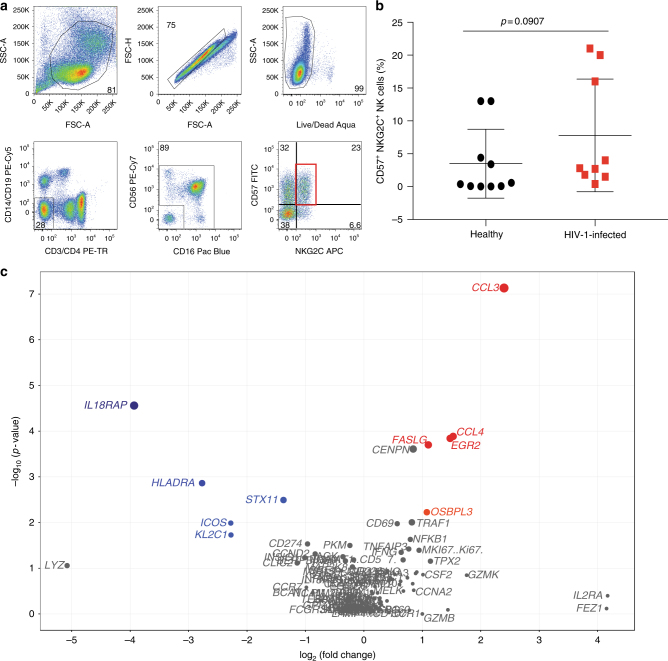
Cell surface expression of NKG2C and gene expression of CD57^+^NKG2C^+^ NK cells in healthy subjects and HIV-1 chronically infected individuals. **a** Flow cytometric gating strategy of sorted CD57^+^NKG2C^+^ NK cells. **b** Graphical analysis of frequency of CD57^+^NKG2C^+^ NK cells in healthy (black) and HIV-1-infected individuals (red). Mann–Whitney test was used to determine significance between frequencies of each group. **c** Volcano plot displaying upregulated (red), downregulated (blue), and stably expressed (gray) pre-selected genes, involved in NK cell-mediated responses to HIV-1 infection. Size of each data point is calculated as −log_10_(*p*-value) × log_2_(FC), *p*-value cutoff *p* < 0.05, fold change cutoff > 2 or <1/2. Transcriptional analysis from six healthy subjects and five HIV-1 chronically infected individuals from one independent experiment is represented

The human NK cell receptor repertoire is skewed toward more highly differentiated CD57^+^NKG2C^+^ NK cells after certain infections, primarily cytomegalovirus (CMV), and after vaccination^35–39^. To assess whether CMV status and a shift in NK cell differentiation contributed to the upregulation of genes observed within the healthy (*n* = 6) and HIV-1-infected (*n* = 5) individuals, we analyzed the targeted gene expression patterns of the CD57^+^NKG2C^+^ NK cell compartment (Fig. 7a and supplementary data 9). The frequency of CD57^+^NKG2C^+^ NK cells between healthy (median with interquartile range = 0.49, 0.048–13) and HIV-1-infected individuals (2.8, 0.4–21) is not significantly different (Wilcoxon rank-sum test, *p* = 0.0907, Fig. 7b). To assess CMV status in healthy and HIV patients we quantified CMV-specific T cells using the IFN-γ ELISpot assay. CMV-specific responses are detected in 3 of 6 healthy individuals and 5 of 5 HIV-infected individuals (supplementary data 11). Several transcripts are significantly upregulated, most notably *CCL3*, *CCL4*, *EGR2*, *FASLG*, and *OSBPL3* with 2.1- to 5.2-fold change increases and *p*-values ranging from 0.00001 to 0.005 (Wilcoxon rank-sum test, Fig. 7c). Among transcripts most significantly downregulated are IL18RAP, HLADRA, STX11, ICOS, and KLRC1 with 2.2- to 3.9-fold change decreases and *p*-values ranging from 0.0002 to 0.02 (Wilcoxon rank-sum test, Fig. 7c). These data suggest that CD57^+^NKG2C^+^ NK cells in chronic HIV-1 infection demonstrate a transcriptional profile strongly linked with overall NK cell activation and inflammation, and moderately linked with ADCC activity. Interestingly and not surprisingly, we observe a significant decrease in transcripts associated with cytokine receptor expression. This highly differentiated subset of NK cells does not differentially express genes that directly overlapped with the transcriptional profile of the overall CD56^dim^CD16^dim/neg^ compartment, however, other genes of NK cell activation are expressed at higher levels compared to uninfected individuals. The presence of additional upregulated genes involved in inflammation and ADCC gives us additional insight into the potential function of the CD57^+^NKG2C^+^ NK cell subset during HIV-1 infection.

**Fig. 8:**
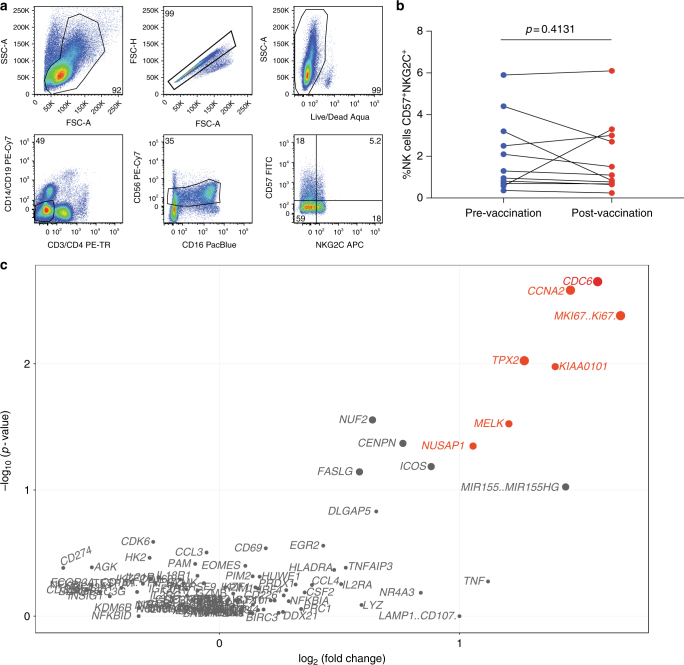
Cell surface expression of NKG2C and gene expression of CD57^+^NKG2C^+^ NK cells in vaccinated individuals. **a** Flow cytometric gating strategy of sorted CD57^+^NKG2C^+^ NK cells. **b** Graphical analysis of frequency of CD57^+^NKG2C^+^ NK cells in vaccinated individual’s pre (blue)- and post (red) vaccination. Wilcoxon matched-pair sign-rank test was used to determine significance between frequencies of each group. **c** Volcano plot displaying upregulated (red) and stably expressed (gray) pre-selected genes, involved in NK cell-mediated responses to MVA vaccination. Size of each data point is calculated as −log_10_(*p*-value) × log_2_(FC), *p*-value cutoff *p* < 0.05, fold change cutoff > 2 or <1/2. Transcriptional analysis from 11 vaccinated individuals and 1 independent experiment is represented

To assess whether a shift in NK cell differentiation contributed to the changes in gene expression observed within the vaccinated individuals (*n* = 11), we analyzed the targeted gene expression patterns of the CD57^+^NKG2C^+^ CD56^dim^CD16^dim/neg^ NK cell compartment (Fig. 8a). The frequency of CD57^+^NKG2C^+^ NK cells in individuals prior to vaccination (1.3, 0.36–5.9) and after vaccination (1.1, 0.24–6.1) was not significantly different (Wilcoxon signed-rank test, *p* = 0.413, Fig. 8b) and the variability of frequency across donors does not correlate with baseline gene expression (CV = 3.3, supplementary Fig. 5 and supplementary data 12). To assess CMV status in vaccinees we quantified CMV-specific T cells using the IFN-γ ICS assay. CMV-specific responses are detected in 7 of 11 vaccinees (supplementary data 13). Thereafter, we examined targeted gene expression of CD57^+^NKG2C^+^ NK cells in these individuals pre- and post vaccination (supplementary data 10). Among transcripts most significantly upregulated after vaccination are *CDC6*, *CCNA2*, *MKI67*, *TPX2*, *KIAA0101*, *MELK*, and *NUSAP1* with 2.07- to 3.18-fold change increases and *p*-values ranging from 0.002 to 0.04 (Wilcoxon signed-rank test, Fig. 8c). Furthermore, the frequency of IFN-γ+ CMV-specific T cells does not correlate to baseline gene expression in CD57^+^NKG2C^+^ NK cells (supplementary Fig. 6) suggesting that CMV status has no impact on the upregulation of genes post vaccination (Fishers exact test, *p* > 0.5). Overall, these data demonstrate that the presence of CD57^+^NKG2C^+^ NK cells could be significantly contributing to the modulated NK cell activity after vaccination and this modulation is associated with the direct cell recognition and cytolysis.

## Discussion

NK cells are innate lymphocytes with enormous phenotypic and functional diversity; however, traditional analysis by flow cytometry offers limited capacity to distinguish the nature of the response. In attempt to gain better resolution of NK cell activity we surveyed the entire transcriptome of in vitro-stimulated NK cells from healthy donors, which revealed common and unique transcriptional signatures of NK cells responding to specific stimuli. To date, limited work has been performed to understand the transcriptional profiles of NK cells after activation^34,40,41^. Here we unmasked transcriptomic fingerprints of NK cell activity in response to models of direct cell recognition, ADCC, and cytokine stimulation in vitro. We then developed a targeted transcriptional panel and applied it to a cohort of HIV-1 chronically infected individuals as well as a cohort of HIV-1-vaccinated individuals, aiming to delineate potential mechanisms of NK cell function within the CD56^dim^CD16^dim/neg^ compartment. We successfully identified a common transcriptional fingerprint associated with overall NK cell activation in HIV-1 chronically infected individuals and a distinctive transcriptional fingerprint corresponding with direct cell cytolysis in vaccinated individuals.

The common upregulated transcriptional signature, determined by whole-transcriptome analysis, include *KDM6B*, *IRF4*, and *TNFAIP3*, and cluster within immune activation pathways. Utilizing this information, we were then able to examine these genes directly ex vivo in HIV-1 chronically untreated individuals, using our targeted transcriptional panel. This data revealed that *KDM6B*, *IRF4*, and *TNFAIP3* have statistically significant higher levels of gene expression compared to normal healthy individuals within the CD56^dim^CD16^dim/neg^ NK cell subset. We also observed statistically significant lower levels of gene expression of *GZMK*, *IL12RB2*, and *LYZ* compared to normal healthy controls. Within upregulated genes, *IRF4* is important in the regulation of IFN-γ in response to infection by virus, and in the regulation of interferon-inducible genes. Cooperative binding of *IRF4* with *BATF* regulates activation of innate and adaptive immune systems in a lymphocyte specific manner^34^. Lysine demethylase 6B (*KMDB6*) works in conjunction with *IRF4* and IFN-γ, which regulates gene expression during inflammation. Collectively, this can account for the common states of activation and inflammation seen during HIV-1 chronic infection^42^. While NK cells are an important antiviral effector population, it is well documented that chronic HIV-1 infection is associated with impaired NK cell function^17,43,44^. Of note, we observed a trend of decreased expression of *LAMP1*, *IL18R1*, and *IL2RA*, genes within the transcriptional profiles of cytotoxicity and cytokine responsiveness. This observation further supports common NK cell dysfunction in chronic infection. It is also important to note, although the expression of these genes was only a trend, the sensitivity and nuances of transcriptional profiling allowed us to detect these changes.

Alteration of the NK cell receptor repertoire has been observed most notably in human CMV infection and is associated with increased NKG2C expression, an activating NK cell receptor that binds HLA-E^38,39,45^. In HIV infection, increased expression of NKG2C has also been reported^46^. Furthermore, the absence of a 16-kb segment of DNA encompassing the *NKG2C* gene, termed absence variation, is associated with increased susceptibility to HIV infection, higher viral set point, and faster disease progression^46^. These data combined indicate NKG2C is important in the defense against HIV infection and progression. CD57, another important marker of NK cell maturation^47^ and functional potential^30^, is enriched on NKG2C^+^ NK cells compared to NKG2C^−^ NK cells^39^. Heath et al. has demonstrated that this NK cell subset is significantly expanded in HIV-infected donors when co-infected with CMV. Therefore, with these previous finding combined we investigated the frequency of CD56^dim^CD16^dim/neg^ NK cells expressing NKG2C and CD57 as well as their gene expression profile within healthy, HIV-1 chronically infected, and vaccinated individuals. Strikingly, within the CD57^+^NKG2C^+^ NK cell subset *CCL3* (MIP-1α), *CCL4* (MIP-1β), *EGR2*, *FASLG*, and *OSBPL3* were upregulated at significant levels. While these genes do not specifically overlap with upregulated genes within the CD56^dim^ subset, they fall within the same common upregulated transcriptional signature of overall NK cell activation, with the exception of OSBPL3, involved in ADCC activity, giving insight to the potential functionality of these highly differentiated cells. Downregulated genes include *IL18RAP*, *HLADRA*, *STX11*, *ICOS*,* and KLRC1*, which fall within the cytokine transcriptional fingerprint. These genes also do not directly overlap with downregulated genes within the CD56^dim^CD16^dim/neg^ subset. Although we cannot discount that CMV status of the healthy and HIV-1-infected individuals, or lack thereof, could be a potential confounder to these results, when assessing variable frequencies of NKG2C^+^CD57^+^ NK cells we did not see a correlation to CMV-specific T cells. A plausible explanation for this is independent expansion of NKG2C^+^CD57^+^ NK cells compared to CD4^+^/CD8^+^ T-cell ratio, suggesting compartmentalization of NK and T cells during CMV infection^39^.

Interestingly, the CD57^+^NKG2C^+^ NK cell population has been associated with a defect in the ability to respond to cytokines^39^. We also see that there is a reduction in an accessory protein that supports IL-18 signaling (*IL18RAP*). These combined data suggest that while not identical, transcriptional activity within the CD56^dim^ and CD57^+^NKG2C^+^ NK cell subsets may contribute to overall activation, inflammation, and ADCC responses in chronic HIV-1 infection.

Subsequently, we continued to apply our targeted transcriptional approach and examine individuals pre- and post vaccination with a live viral vector, MVA-CMDR HIV-1 vaccine. MVA is a replication-deficient poxvirus that can provide an efficient platform for the delivery of HIV-1 antigens to stimulate adaptive immunity^48,49^. Kirwan et al.^50^ has shown that vaccinia virus infection is associated with decreased MHC class I expression on the target cells leaving them susceptible to NK-mediated lysis. Chisholm et al.^51^ showed that the marked increase in vaccinia virus-infected target cell susceptibility to NK cell lysis is not based on downregulation of MHC-I but increased ligands for the natural cytotoxicity receptors. It is important to note that most studies have examined response to vaccinia virus, or other orthopox viruses, but not the lab-attenuated virus strain. Other studies have looked at innate and adaptive cell mobilization within hours and days post vaccination, which was not possible in this study. However, Zak et al.^52^ demonstrated that in response to Ad5 vaccination there is a significant efflux of CD56dim NK cells 24 h post vaccination. In addition, Marquadt et al.^53^ showed peak activation and functionality of NK cells at days 6 and 10, respectively, in response to yellow fever virus vaccine. Due to this information and based on sample availability we chose to look at transcriptional signatures of NK cell activation 7 days post vaccination compared to baseline.

Intriguingly, 7 days post vaccination, CD56^dim^CD16^dim/neg^ NK cells displayed increased expression of NUSAP1, MKI67, TPX2, KIAA0101, CCNA2, MELK, and DLGAP5 compared to pre-vaccination samples. These data corroborate the HTA data of transcripts uniquely upregulated in K562 stimulation, a transcriptional fingerprint of direct cell recognition and cytotoxicity. These genes also cluster within the microtubule cytoskeleton pathway as well as cellular assembly and organization pathways. Particularly, NUSAP1 encodes a microtubule-associated protein with the capacity to bundle and stabilize microtubules, which suggests that changes within cytoskeleton organization may affect the polarization or release of lytic molecules prior to killing of infected targets^41^. Intriguingly, within the CD57^+^NKG2C^+^ NK cell subset *CDC6*, *CCNA2*, *MKI67*, *KIAA0101*, *TPX2*, *MELK* and *NUSAP1* were significantly upregulated. This represents a 71% overlap with genes upregulated within the CD56^dim^CD16^dim/neg^ NK cell compartment, suggesting the transcriptional activity of CD57^+^NKG2C^+^ NK cells make a substantial contribution to NK cells involved in direct cell mechanisms, post vaccination. Our data support a model whereby MVA stimulates a robust and potentially cytotoxic NK cell response, which may contribute to its capacity as a potent immunogen and thus impact subtle differences in host immune responses.

ADCC has been shown to be a correlate of protection from HIV-1 infection and is known to be partially responsible for controlling HIV-1 viral replication after infection, resulting in slower disease progression^54–57^. The transcriptional profile of ADCC, identified in the HTA analysis, includes genes *BCL2*, *ENO1*, *GPI*, and *OSBPL3*. *BCL2* suppresses apoptosis by inhibiting caspase activity, thereby keeping the NK cells alive to continue performing its effector function. *ENO1* and *GPI* encode multifunctional enzymes that stimulate immunoglobulin production and could potentially have a positive effect on B cells producing IgG^58^. *OSBPL3* encodes a protein involved in the organization of the actin cytoskeleton, and also may be involved in the release of lytic molecules^41^. Together, these genes cluster within the immune response pathway and can be used as an identifier of NK cells mediating ADCC. Much to our surprise, when interrogating the total CD56^dim^CD16^dim/neg^ NK cell compartment in HIV-1 chronic infection we did not see any active transcriptional signatures associated with ADCC. This may reflect the NK cell dysfunction and exhaustion observed in chronic untreated HIV-1 infection or could be reflective of the disease state of the donors included in this analysis^59^. Of note, when narrowing our interrogation to the CD57^+^NKG2C^+^ NK cell subset, *OSBPL3*, a gene within the ADCC transcriptional profile, was upregulated at statistically significant levels. This cell subset could potentially have ADCC functionality but is not a significant contributor to ADCC activity within the overall CD56^dim^CD16^dim/neg^ NK cell compartment.

Our study of chronic and vaccine study participants was limited by bulk-cell analysis. This may account for the failure to identify an ADCC transcriptional profile within these groups. We are also limited by the fact that in vitro models of ADCC, such as the GP120-coated CEM.NKR.CCR5 cells, do not recapture the envelope conformation that occurs in vivo^60^. This could also account for the failure to identify an ADCC transcriptional profile within these groups. Therefore, with similar rationale of adding the P815 model of ADCC used in the whole-transcriptome analysis, we tested a third model of ADCC using plate-bound CD16 as a stimulation condition in the targeted gene expression analysis (supplementary Figure 3).

As with in vitro models of ADCC, K562 stimulation is not the only model of direct cell activation of NK cells. Accordingly, we tested six additional stimulatory cell lines and analyzed targeted gene expression patterns, post stimulation. Flow cytometric analysis showed decreased MFI in CD16 expression across all stimulation conditions, albeit at varying levels (supplementary Figure 4A). We observed different patterns of gene expression across all stimulation conditions, and at varying expression levels, demonstrating that numerous models of direct cell activation and ADCC can used to identify transcriptional fingerprints of responding NK cells (supplementary Figure 4B).

When further interrogating gene expression in healthy donors we recognized that technology yields different results at the single-cell level and bulk-cell levels. There were differences between the 96-gene multiplex system (Fluidigm) and whole-transcriptome analysis (HTA, Affymetrix Technologies). We concluded that in some cases exploration at the single-cell level was necessary to unmask unique transcriptional profiles. While HTA analysis cast a broad net to help identify genes in NK cell function, single-cell analysis revealed more information in regards to NK cell transcriptional profiles. Future studies should incorporate examining NK cells at the single-cell level because of their heterogeneity and the resolution that single-cell analysis provides. It is important to note that this analysis could be limited due to sorting and examination of the CD16^dim/negative^ population and excluding CD56^bright^ and CD56^negative^ populations. Our results could also be biased due to in vitro stimulation, however this is an integrative approach and hypothesis-generating procedure.

Overall, our results directly demonstrate that NK cells display unique transcriptomic fingerprints involved in various pathways and mechanisms contingent on stimulus. These transcriptomic fingerprints can be used to interrogate cell function within numerous NK cell subsets, directly ex vivo, in various disease states and highlight the potential for NK cell-targeted treatments to improve immune responses, in the context of vaccination or therapeutic interventions during infection.

## Methods

### Study participants and cell lines

Normal, healthy, individuals’ protocol (RV229) and treatment naive chronically HIV-1-infected individuals’ (RV149) cryopreserved leukapheresed peripheral blood mononuclear cells (PBMC) were used for this study. In addition, samples from a Phase I Study of the Safety and Immunogenicity of PennVaxG DNA (env and gag) by MVA-CMDR (HIV-1 CM235 env/CM240 gag/pol) boost in healthy, HIV-uninfected adults (RV262) were used. Eleven donors were selected and two time points were chosen, pre-vaccination and 7 days post MVA boost, to determine gene expression in NK cells^61^. All individuals participating in this study provided written informed consent. Ethical approval was obtained from institutional review boards in each country, including the Human Subjects Protection Branch, Walter Reed Army Institute of Research; Ethical Review Committee for Research in Human Subjects, Ministry of Public Health, Thailand; and Siriraj Institutional Review Broad, Faculty of Medicine, Siriraj Hospital Mahidol University. All experimental procedures were performed in Biosafety Level 2 or higher laboratories.

The CEM.NKR.CCR5 cell line was obtained through the NIH AIDS Reagent Program, Division of AIDS, NIAID, NIH. The *Mus musculus*, mouse mastocytoma cell line, P815 (TIB-64) was obtained from the American Type Culture Collection (ATCC; Manassas, VA)^11^. The chronic myelogenous leukemia lymphoblast cell line, K562 (CCL-243), was obtained from the ATCC. The HLA-A-, -B-, and -C-negative mutant B-lymphoblastoid cell line 721.221 and 721.221 transfectant, expressing a single HLA class I allele (Cw04) were kindly provided by John Coligan (NIAID, NIH)^62,63^. The human monocyte cell line U-937 (CRL-1593.2) was obtained from the ATCC. The human T-lymphoblastoid cell line A3R5 were obtained courtesy of Dr. Robert McLinden^64^. Determination of CMV status within all healthy and HIV-1 chronically infected individuals was determined by testing for CMV-specific T cells via IFN-γ ELISpot using CMVpp65 as a stimulant. Post-assay IFN-γ SFCs/10^6^ were detected and calculated using the Immunospot CTL reader (CTL, Cleveland, OH). CMV status for vaccinees were determined by testing for CMV-specific IFN-γ+ T cells via flow cytometry using CMVpp65 as a stimulant. Cells were stained and run on a FACS LSRII (BD Biosciences, San Jose, CA).

### Polychromatic flow cytometry

PBMC from four healthy donors in four repeated experiments were stained as previously described^23,65^. Briefly, three polychromatic flow cytometry-based assays were used to measure NK cell function and phenotypic staining for cell sorting.

### Function assay

A six-function assay was performed from PBMCs, from four healthy donors, stimulated for 6 h with K562 cells, gp120-coated CEM.NKR.CCR5 cells in the presence of HIV-IG, IL-12/IL-18, or in the absence of stimulation. PBMC were thawed in 20% fetal bovine serum-containing media supplemented with Benzonase nuclease, and counts and viabilities were performed using Guava ViaCount reagent and the Guava PCA (Guava Technologies, Hayward, CA). Cells were then stimulated in the presence of CD107a FITC (H4A3, dilution 1/400, Becton Dickinson (BD, San Jose, CA)), in 96-well polypropylene U-bottom plates at 5% CO2 at 37 °C with 90% relative humidity for 6 h. After 2 h of stimulation, brefeldin A and monensin were added to inhibit protein transport. One million cells were used per stimulation condition. Cells were washed and stained with Aqua Live/Dead stain (Molecular Probes, Eugene, OR). Cells were washed, blocked using normal mouse IgG (Caltag, Eugene, OR), and surface-stained for CD14 (Tuk4, dilution 1/160, Invitrogen) and CD19 PE-Cy5 (SJ25-C1, dilution 1/40, Invitrogen), CD56 PE-Cy7 (NCAM16.2, dilution 1/320, BD), CD16 PacBlue (3G8, dilution 1/80, BD), and CD57 APC (HCD57, dilution 1/10, Biolegend). Cells were washed, fixed in 2% formaldehyde for 15 min, and washed again. Cells were then permeabilized using Perm/Wash (BD) and stained intracellularly with CD3 APC-Cy7 (SP34-2, dilution 1/160, BD), CD4 APC-H7 (SK3, dilution 1/40, BD), CD8 BV785 (RPA-T8, dilution 1/40, Biolegend), IFN-γ BV711 (4S.B3, dilution 1/160, Biolegend), TNF BV650 (Mab11, dilution 1/80, Biolegend), Perforin PE (B-D48, dilution 1/20, eBiosciences), Granzyme B PE CF594 (GB11, dilution 1/160, BD), and Eomesodermin PerCP eFluor710 (Dan11mag, dilution 1/20, eBiosciences). Cells were washed and run on a FACS LSRII (BD Biosciences).

### Cell sorting

Two 12-color panels were used to phenotype, surface stain, and sort CD56^dim/neg^ and CD57^+^NKG2C^+^ NK cells from 9 healthy donors, 7 HIV-1 chronically infected individuals, and 11 vaccinees. PBMC were thawed in 20% fetal bovine serum-containing media supplemented with Benzonase nuclease, and counts and viabilities were performed using Guava ViaCount reagent and the Guava PCA (Guava Technologies). Cells were washed and stained with Aqua Live/Dead stain (Molecular Probes). Cells were washed and blocked using normal mouse IgG (Caltag). For the CD56^dim^CD16^dim/neg^ phenotyping panel PBMC were surface-stained for CD57 FITC (HCD57, dilution 1/10, BD), KIR2DL1/DS1 (HP-MA4, dilution 1/80, e-Bioscience), CD16 Pacific Blue (3G8, dilution 1/80, BD), CD62L BV605 (DREG56, dilution 1/20, Biolegend), HLA-DR BV650 (G46-6, dilution 1/20, Biolegend), and α4β7 AlexaFluor647 (Act-1, NIH-ARRRP, AlexaFluor647 conjugation kit, Invitrogen, conjugated antibody used at 1/100 dilution). Post surface staining cells were washed, filtered, and CD56^dim^CD16^dim/neg^ cells sorted on a FACS ARIA (BD Biosciences). For the CD57^+^NKG2C^+^ phenotyping panel PBMC were surfaced-stained for NKG2C APC (FAB138A, 1/50 dilution, R&D), KIR3DL1 Alexa Fluor700 (DX9, dilution 1/160, Biolegend), CD8 APC-H7 (SK1, dilution 1/80, BD), KIR2DL2/3/DS2 PE (DX27, dilution 1/10, BD), CD3 PE-Texas Red (S4.1, dilution 1/160, Invitrogen), CD4 ECD (SFCI12T4D11, dilution 1/40, Beckman Coulter), CD14 PE-Cy5 (Tuk4, dilution 1/640, Invitrogen), CD19 PE-Cy5 (SJ25-C1, dilution 1/40, Invitrogen), and CD56 PE-Cy7 (NCAM16.2, dilution 1/320, BD). Post surface staining cells were washed, filtered, and CD57^+^NKG2C^+^ cells sorted on a FACS ARIA (BD Biosciences).

### RNA extraction

RNA from CD56^dim^CD16^dim/neg^ and CD57^+^NKG2C^+^ sorted cells were extracted using RNAqueous Micro kit (Ambion, Thermo Fisher Scientific, catalog number AM1931) according to the manufacturer’s instructions. Extracted RNA was measured and checked for concentration, yield, and purity on a Nanodrop spectrophotometer (Thermo Fisher Scientific). RNA concentrations were normalized before multiplexed assays were run.

### Transcriptome analyses

PBMC from six healthy donors in two separate experiments were stimulated for 6 h with K562 cells, gp120-coated CEM.NKR.CCR5 cells in the presence of HIV-IG, P815 cells plus P815-specific Abs, IL-12/IL-18, or medium alone. An effector-to-target ratio of 10:1 was used for each cell line. One million cells were used per stimulation condition. Post stimulation cells were washed and stained with Aqua Live/Dead stain for 30 min at room temperature (Molecular Probes). Cells were then washed, blocked using normal mouse IgG (Caltag) and surface-stained with the CD56^dim^CD16^dim/neg^ phenotyping panel, as described above, and sorted. RNA was extracted from sorted cells as described above. Extracted RNA from bulk CD56^dim^CD16^dim/neg^ NK cells from six healthy individuals was sent to State University of New York Molecular Analysis Core. To ensure uniform coverage of the transcriptome, Affymetrix Gene Chip HTA 2.0 was used to determine gene expression across the three stimulation conditions. The CEL files generated by these arrays are analyzed with Affymetrix Expression Console™ Software (version 1.3) and Affymetrix^®^ Transcriptome Analysis Console (TAC) Software, which enabled visualization of the data and expression changes at the gene and exon level.

### Selection of targeted gene expression panel

96-transcript panel was selected based on fold change −2 ≥ 2 compared to unstimulated controls, *p*-value ≤ 0.05, and FDR value ≤ 0.1. After reaching fold change and statistical criteria, all transcripts were verified by gene accession numbers. Genes were then quality controlled to determine qualification for expression down to the single-cell level, ensuring sensitive detection of gene expression within a heterogeneous bulk population. The primer qualification evaluates efficient amplification, linearity, and multiplexing capability^66^. Qualifications were performed with 12-point, twofold dilution series, replicated eight times across a plate. Expression threshold (ET) values were plotted against the RNA quantity and every replicate, 12-dilution series was divided into 7 sequential segments, each comprising 6 consecutive dilutions. Segments were evaluated in a step-wise, iterative manner, beginning with the lowest RNA amount. For each segment, the linear least-squares correlation between the logarithm of the RNA amount and amplification cycles (ET; 40-CT) was determined. The segment passed if the correlation was strong (*r*^2^ > 0.99), and if the slope of this fit indicated a linear relationship between signal (2ET) and RNA. If amplification was 100% efficient, the slope would equal 3.32 (1/log102). The efficiency (*E*) was calculated from the formula: *E* = 101/slope − 1. We accepted slopes between 3.1 and 3.6 (*E* = 90–110%)^66^. Unique transcripts that met all four statistical criteria and passed qualification were included in the final 96-targeted gene expression panels to represent genes related to each NK cell mechanism as well as common genes across all three mechanisms.

### Bulk- and single-cell transcriptional analysis panel NK1.0

CD56^dim^CD16^dim/neg^ NK cells from four healthy individuals (~1000 cells/well or single cell/well) were sorted (SuperScript III Reverse Transcriptase/Platinum Taq Mix, Cells Direct 2× Reaction Mix, Invitrogen). Reverse transcription and specific transcript amplification were performed using a thermocycler (Applied Biosystems Gene Amp PCR System 9700) as follows: 50 °C for 15 min; 95 °C for 2 min; then 95 °C for 15 s; and 60 °C for 30 s for 18 cycles. The amplified cDNA was loaded into Biomark 96.96 Dynamic Array chips using the Nanoflex IFC controller (Fluidigm). This microfluidic platform was then used to conduct single-cell quantitative PCR (qPCR) in nanoliter reaction volumes. Threshold cycle (CT), as a measurement of relative fluorescence intensity, was extracted from the BioMark Real-Time PCR Analysis software. Data were analyzed as described in Statistical analysis section.

### Bulk analysis using targeted transcriptional panel NK2.0

Extracted RNA from bulk sorted CD56^dim^CD16^dim/neg^ NK cells from 9 healthy, 7 HIV chronically infected, and 11 vaccine recipients was plated into 96-well skirted plate containing 10 μl of reaction buffer, cDNA was generated and qPCR was performed as described above. CD56^dim^CD16^dim/neg^ and CD57^+^NKG2C^+^NK cells from 9 healthy, 7 HIV chronically infected, and 11 vaccine recipients were sorted into 96-well skirted plate (~1000 cells/well) containing 10 μl of reaction buffer, cDNA was generated and qPCR was performed as described above.

### Gene Set Enrichment Analysis

Gene Set Enrichment Analysis (GSEA) Broad Institute of MIT and Harvard is a computational method that determines whether an a priori defined set of genes shows statistically significant, concordant differences between two biological states. The leading edge subset of a gene set is the subset of members that contribute most to the enrichment score (ES)^67^. For a positive ES, the leading edge subset is the set of members that appear in the ranked list prior to the peak score. For a negative ES, it is the set of members that appear subsequent to the peak score. The leading edge subset can be interpreted as the core that accounts for the gene set’s enrichment signal. GSEA software was used as previously described to determine pathway analysis^67,68^.

### Statistical analysis

All flow data were analyzed in FlowJo LLC, Treestar Inc. (Ashland, OR), and then permutation tests were performed to determine statistical significance in comparison of pie charts generated in Spice software (Bethesda, MD)^69^. Volcano plots of the HTA data were generated using an affymetrix data table containing adjusted *p*-values and fold change pre-calculated by Affymetrix^®^ TAC Software. Affymetrix data are normalized with R/bioconductor packages; limma, affy. Red dots are fold change > 2, *p*-value < 0.05, and blue dots are fold change < 1/2, *p*-value < 0.05. R with bioconductor packages; ggplots, ggthemes, colorspace were used to make plots. PCA was performed with R basic function, prcomp method, and results are visualized using R plotly bioconductor package. Principle components 1, 2, and 3 were used to make the three-dimensional plots. GSEA was performed with Xcode programming and bioconductor/R. Affymetrix files were normalized into Gene Cluster Textile format (.gct), which is suitable for GSEA analysis. NES scores of each gene were used to make a heat map. Heat map was made by gplots package in R/Bioconductor. Fluidigm data were normalized with limma bioconductor package with gplots. RColorBrewer package were used to make heatmaps. ET values were calculated based on CT values (40-CT) obtained from the qPCR data and plotted using Prism Software. Fold change was determined by comparison of ET values. *p*-values were calculated using unpaired Wilcoxon rank-sum tests for gene expression in healthy donors and HIV-1 chronically infected donors, and paired Wilcoxon signed-rank tests for vaccinees. Volcano plots were generated using R with bioconductor packages; ggplots, ggthemes, colorspace. Fold change and *p*-values are displayed as log_2_ and negative log_10_ values, respectively. Spearman rank correlation was used for all correlation scatter plots. Fisher’s exact test was used to determine if CMV status had an impact on the upregulation of genes post vaccination.

### Data availability

HTA data that support the findings of this study have been deposited in NCBI’s Gene Expression Omnibus and are accessible through GEO Series accession number GSE110446.

The authors declare that the data supporting the findings of this study are available within the article and its supplementary information files, or are available upon reasonable requests to the authors.

## Electronic supplementary material

Supplementary Information(PDF 1206 kb)

Description of Additional Supplementary Files(PDF 175 kb)

Supplementary Data 1(XLSX 26 kb)

Supplementary Data 2(XLSX 50 kb)

Supplementary Data 3(XLSX 11 kb)

Supplementary Data 4(XLSX 34 kb)

Supplementary Data 5(XLSX 14 kb)

Supplementary Data 6(XLSX 46 kb)

Supplementary Data 7(XLSX 54 kb)

Supplementary Data 8(XLSX 50 kb)

Supplementary Data 9(XLSX 12 kb)

Supplementary Data 10(XLSX 55 kb)

Supplementary Data 11(XLSX 44 kb)

Supplementary Data 12(XLSX 43 kb)

Supplementary Data 13(XLSX 41 kb)
