# Supplementary material for: Transcriptomic signatures of NK cells suggest impaired responsiveness in HIV-1 infection and increased activity post-vaccination
Source: Nat Commun. 2018 Mar 23;9:1212. doi: 10.1038/s41467-018-03618-w (PMC5865158; doi:10.1038/s41467-018-03618-w)
Supplement: Supplementary file 1 — Supplementary Information(PDF 1206 kb) [file 41467_2018_3618_MOESM1_ESM.pdf]

Supplementary Information

Transcriptomic signatures of NK cells suggest impaired responsiveness in HIV-1 infection and increased activity post-vaccination.

Costanzo et.al

Supplemental Figure 1:

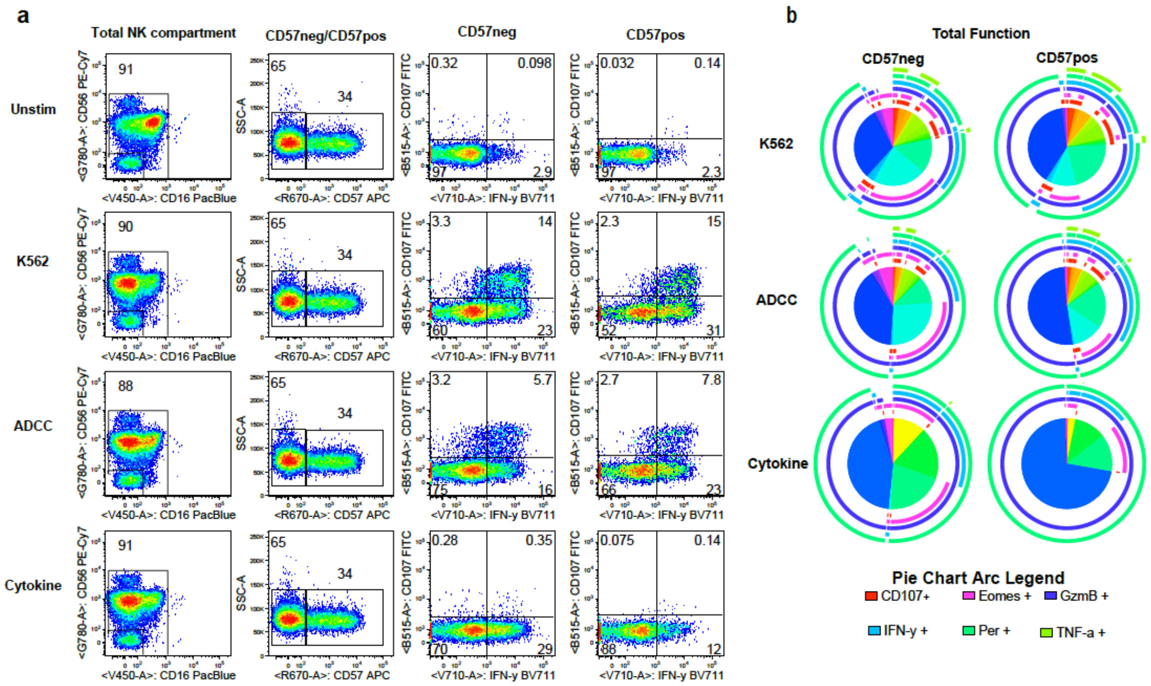

Supplemental Figure 1. **Functional comparison of responding CD57- and CD57+ NK cells across multiple stimulation conditions**. (a) First column- CD56 and CD16 are used to identify NK cells and changes in CD16 expression across stimulation conditions. Second column- Black gates identify CD57- and CD57+ cells within the total NK compartment. Third and fourth columns- CD107a and IFN-γ expression within the CD57- and CD57+ NK cells, respectively. (b) Pie charts of classic boolean analysis of five functional markers and one transcription factor measured (CD107a, IFN-γ, TNF-a, Granzyme B, Perforin and Eomesodermin). Comparison of pies and permutation tests were performed to determine statistical significance between pies. There was no statistical significance between pie charts across the stimulation conditions.

Supplemental Figure 2:

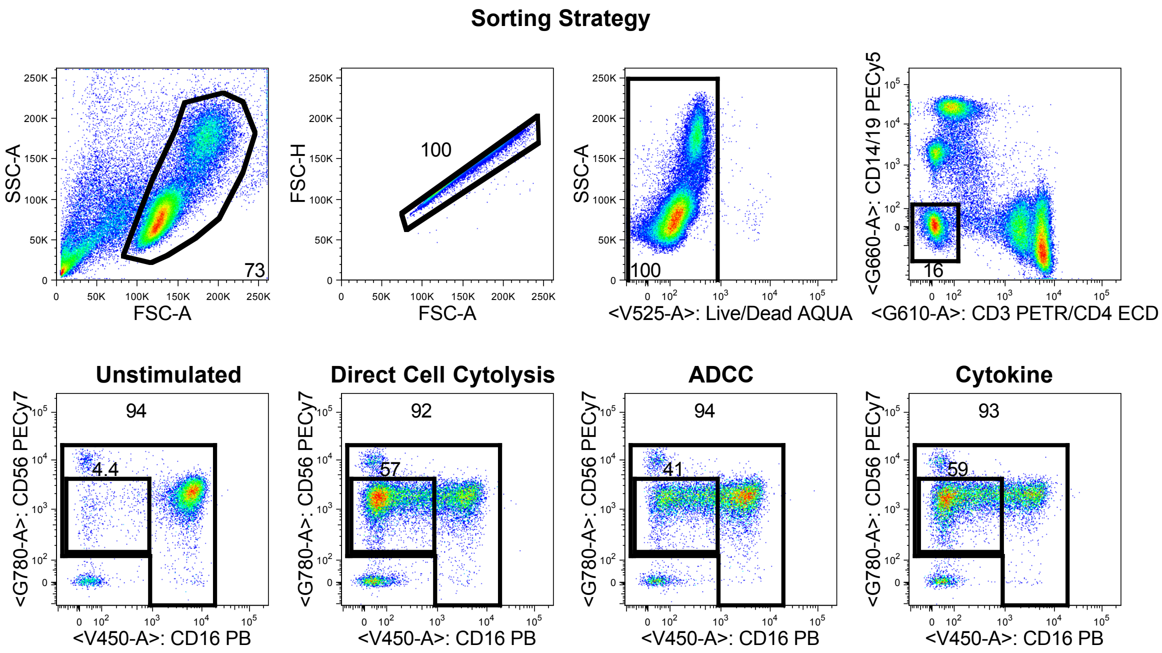

Supplemental Figure 2. Gating and sorting strategy of functional NK cells used for gene expression analysis. Lineage marker gating to identify NK cells via expression of CD56 and CD16 under various stimulation conditions. The rectangular gate capturing cells with decreased CD16 expression within the CD56 dim compartment identifies sorted cells.

Supplemental Figure 3:

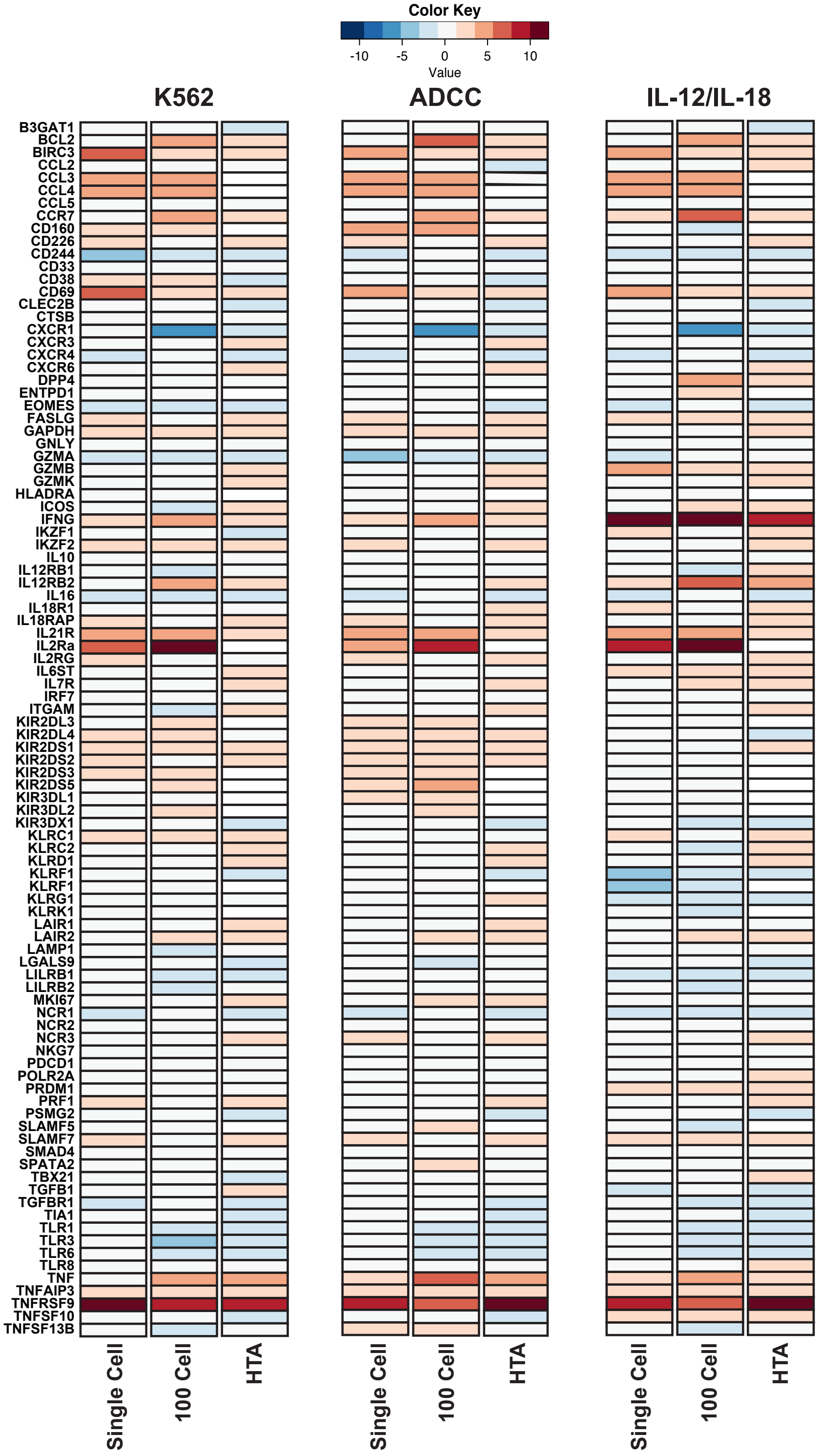

Supplemental Figure 3. **Gene expression of responding NK cells at single, 100 cell and whole transcriptome levels.** (A) Heatmap of gene expression of 96 pre-selected genes involved in NK responses across stimulation conditions, ranked by fold change at the single, 100 and bulk cell levels. Data represented is from three donors and three stimulation conditions, K562, ADCC and IL-12/IL-18. Expression threshold (ET) values were calculated based on CT values (40-CT) obtained from the qPCR data and fold change was determined by comparison of ET values.

Supplemental Figure 4:

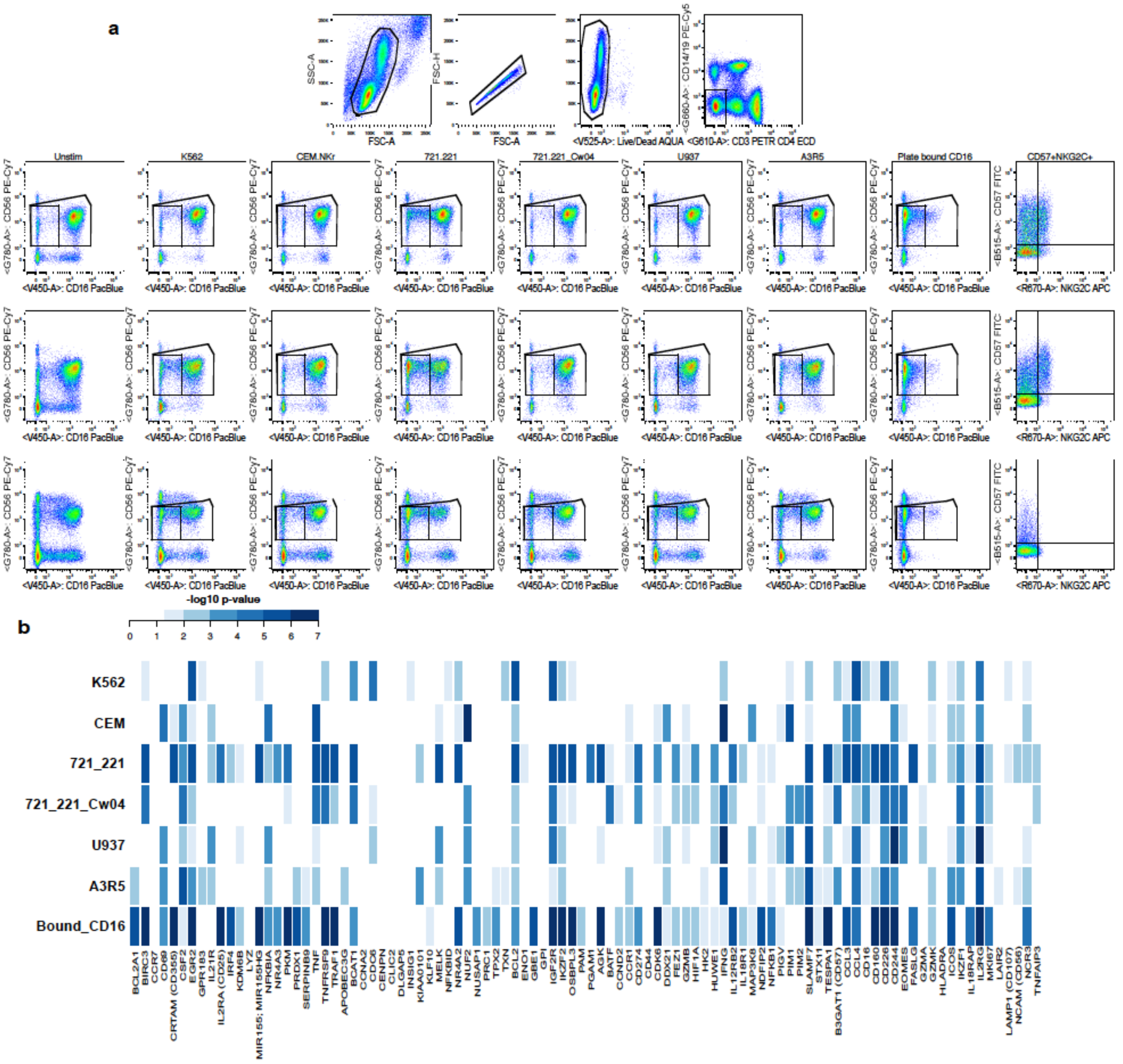

Supplemental Figure 4. **Flow cytometric analysis and gene expression of responding NK cells across 7 stimulation conditions modeling direct cell recognition and ADCC.** (A) Lineage marker gating to identify NK cells via expression of CD56 and CD16 under various stimulation conditions. The rectangular gate capturing cells with decreased CD16 expression within the CD56 dim compartment identifies sorted cells. (B) Heatmap of gene expression of 96 pre-selected genes involved in NK responses across stimulation conditions, ranked by p-value. Data represented is from three donors and seven stimulation conditions.

Supplemental Figure 5:

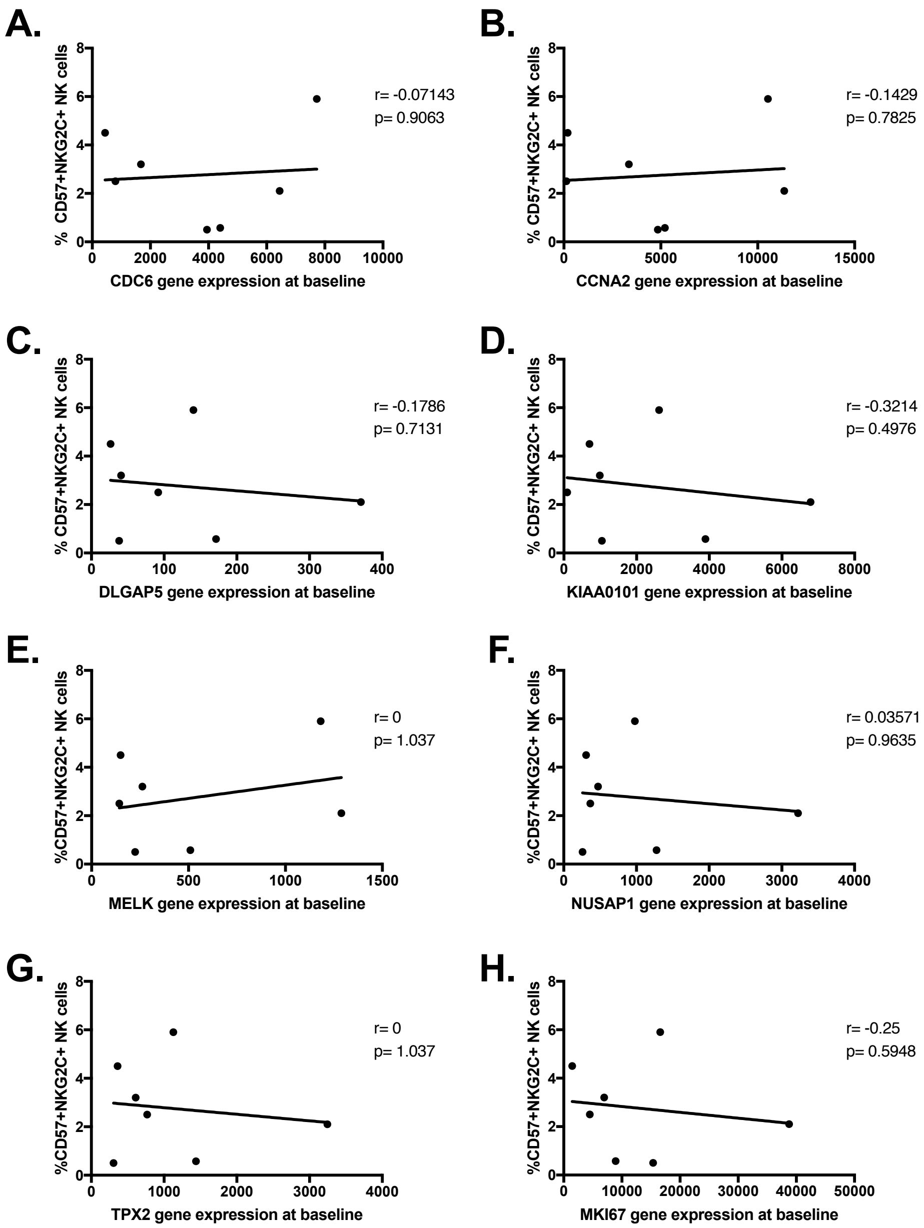

Supplemental Figure 5. **Correlation of CD57+NKG2C+ NK cells and baseline gene expression in vaccinees.** (A-H) Scatterplots representing correlation between the frequency of CD57+NKG2C+ NK cells and baseline gene expression of genes up-regulated post vaccination. Data represented is from 7 donors using Spearman rank correlation.

Supplemental Figure 6:

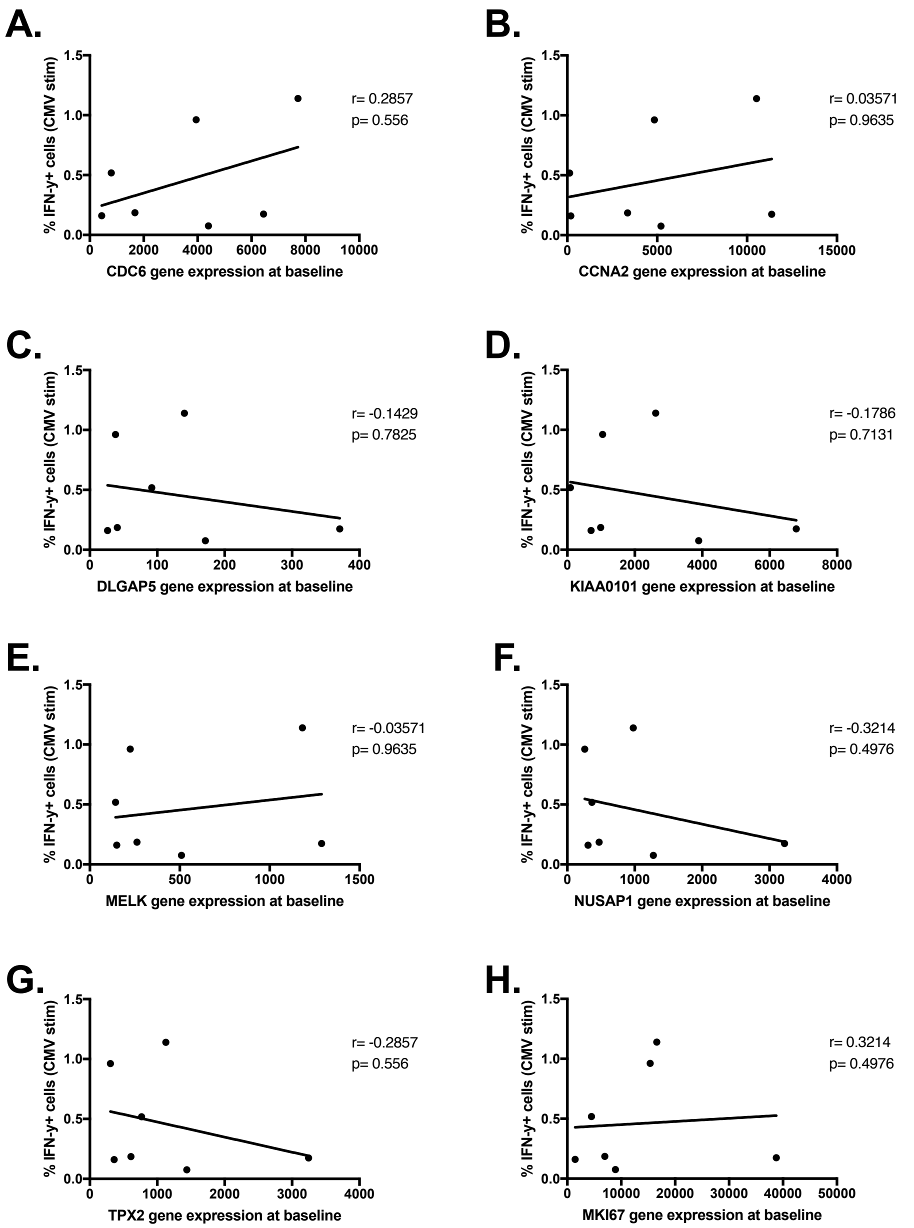

Supplemental Figure 6. **Correlation of IFN-γ^{+} CMV-specific T cells and baseline gene expression in vaccinees.** (A-H) Scatterplots representing correlation between the frequency of IFN-γ^{+} CMV-specific T cells and baseline gene expression of genes up-regulated post vaccination in CD57+NKG2C+ NK cells. Data represented is from 7 donors using Spearman rank correlation.
