# Supplementary material for: Transcriptomic signatures of NK cells suggest impaired responsiveness in HIV-1 infection and increased activity post-vaccination
Source: Nat Commun. 2018 Mar 23;9:1212. doi: 10.1038/s41467-018-03618-w (PMC5865158; doi:10.1038/s41467-018-03618-w)
Supplement: Supplementary file 2 — Description of Additional Supplementary Files(PDF 175 kb) [file 41467_2018_3618_MOESM2_ESM.pdf]

Description of Additional Supplementary Files

File Name: Supplementary Data 1
Description: List of up-regulated genes in HTA data.

File Name: Supplementary Data 2
Description: List of down-regulated genes in HTA data.

File Name: Supplementary Data 3
Description: List of up-regulated genes in ADCC and p815 stimulations in HTA data.

File Name: Supplementary Data 4
Description: List of pathways, NES scores and FDR-q values from GSEA.

File Name: Supplementary Data 5
Description: List of genes and catalogue numbers in NK1.0 targeted gene expression panel.

File Name: Supplementary Data 6
Description: List of genes and catalogue numbers in NK2.0 targeted gene expression panel.

File Name: Supplementary Data 7
Description: Gene list, p-values and fold change from data in volcano plot Figure 5a.

File Name: Supplementary Data 8
Description: Gene list, p-values and fold change from data in volcano plot Figure 6a.

File Name: Supplementary Data 9
Description: Gene list, p-values and fold change from data in volcano plot Figure 7c.

File Name: Supplementary Data 10
Description: Gene list, p-values and fold change from data in volcano plot Figure 8c.

File Name: Supplementary Data 11
Description: Quantification of CMV-specific T-cells via ELISpot.

File Name: Supplementary Data 12
Description: Frequency and cross variance of CD57+NKG2C+ NK cells in vaccinees.

File Name: Supplementary Data 13
Description: Quantification of CMV-specific T-cells via flow cytometry.
